# Low‐Power Short‐Pulse‐Width Fractional Microneedle Radiofrequency Relieves LL37‐Induced Rosacea‐Like Skin Inflammation

**DOI:** 10.1111/jocd.70727

**Published:** 2026-02-09

**Authors:** Zhiyi Xu, Siqi Shen, Jingting Zhao, Jing Wang, Xinlan Wang, Li Gu, Shu Zhou, Jing Zhao, Liqun Gu, Lin Chen, Bingrong Zhou, Hui Hua

**Affiliations:** ^1^ Department of Dermatology Nantong Third People's Hospital, Affiliated Nantong Hospital 3 of Nantong University Nantong China; ^2^ Medical School of Nantong University Nantong China; ^3^ Nantong Institute of Liver Diseases Nantong Third People's Hospital, Affiliated Nantong Hospital 3 of Nantong University Nantong China; ^4^ Department of Dermatology The First Affiliated Hospital of Nanjing Medical, University Nanjing China

**Keywords:** angiogenesis, erythema, FMR, inflammation, LL37, rosacea

## Abstract

**Background:**

Refractory rosacea can be effectively treated with fractional microneedle radiofrequency (FMR), but its optimal parameters need confirmation.

**Objective:**

To explore the optimal parameters of FMR in treating rosacea‐like dermatitis and the underlying mechanisms.

**Methods:**

A rosacea‐like dermatitis mouse model was intervened with FMR of varying pulse energy and width. By assessing the severity of erythema and measuring erythema area, optimal parameters of FMR to treat rosacea‐like dermatitis in mice were determined. Pathological staining was performed to examine the infiltration of inflammatory cells and CD31^+^ microvessels. Expression levels of pro‐inflammatory factors were detected by qRT‐PCR. The involvement of the NF‐κB signaling pathway and its downstream mediators (IL‐1β, IL‐6, TNF‐α) in mice treated with low‐power short‐pulse‐width fractional microneedle radiofrequency (LS‐FMR) was detected using Western blotting. In a cohort of 20 patients with erythematotelangiectatic rosacea (ETR) and managed by one session of FMR treatment, therapeutic efficacy was assessed by Multispectral skin analysis system at 3 months of follow‐up.

**Results:**

LS‐FMR at energy levels of 1, 2, 3 and 4 W and pulse width of 20 ms alleviated the severity of erythema and narrowed the erythema area in LL37‐induced mice. It significantly inhibited the infiltration of mast cells and CD4+ T cells, polarization of CD4+ T cells to Th1/Th17 cells, angiogenesis, the activation of the NF‐κB signaling pathway, as well as the expression of downstream inflammatory cytokines (IL‐1β, IL‐6, TNF‐α). In a cohort of 20 ETR patients, just one session of FMR treatment significantly alleviated erythema at 3 months of follow‐up, without obvious adverse events.

**Conclusion:**

LS‐FMR is a promising approach to treat rosacea by suppressing skin immune responses and angiogenesis.

**Trial Registration:**

Clinical trial number: Not applicable

## 1 Introduction

Rosacea, a chronic inflammatory skin disease mainly affecting the face's central areas, features capillary dilation, recurrent flushing, erythema, and inflammatory papules/pustules [1]. Rosacea typically begins at 30–50 years, cycles between flare‐ups and remissions [2], affects 5.5% of adults globally with a higher rate in women, leading to a high tendency to negative emotions and low quality of life [3].

Immune dysregulation and neurovascular abnormalities are crucial in rosacea pathogenesis [4, 5]. Toll‐like receptor 2 (TLR2) in keratinocytes is upregulated in response to the activation of the innate immune system, which in turn activates kallikrein‐related peptidase 5 (KLK5) into biologically active LL37 [6]. Overexpression of LL37, vital for rosacea development, enhances inflammation by boosting pro‐inflammatory cytokines and NF‐κB signaling [7, 8]. In addition, innate immune cells (such as mast cells) and adaptive immune cells (such as CD4+ T cells) are also closely associated with the pathogenesis of rosacea [9, 10]. Transient receptor potential cation channel subfamily V (TRPV) is found upregulated in rosacea patients and is responsible for skin neurogenic symptoms like burning sensations, tingling, and itching [11, 12]. Currently, LL37 injection is a common methodology to create in vivo rosacea models due to its ability to induce rosacea‐like lesions [13].

How to treat rosacea is challenging. Monotherapy is insufficient to maintain disease stability and prevent relapses, and a long‐term medication enhances the risk of adverse events [14]. Photoelectric therapies, like Intense Pulsed Light (IPL) and Pulsed Dye Laser (PDL), target hemoglobin to reduce vascular dilation and erythema [15, 16]. However, IPL and PDL have been vilified by the poor outcomes of treating deep‐seated skin inflammation and vascular lesions [17]. Studies have shown that 5‐Aminolevulinic acid photodynamic therapy (ALA‐PDT) has demonstrated potential value in the treatment of rosacea. However, the strict postoperative photoprotection requirements of this therapy reduce patient treatment compliance, and its relatively high long‐term recurrence rate limits its prospects for clinical application [18]. Micro‐invasive photoelectric treatments, such as FMR, show better results in rosacea with sebaceous gland hypertrophy and tissue proliferation [19]. Fractional microneedle radiofrequency (FMR), as a minimally invasive cosmetic procedure, can deliver radiofrequency energy directly to the dermis via microneedle arrays, but may cause minimal injury to the epidermis [20]. FMR shrinks sebaceous glands and stimulates collagen generation and remodeling, thus improving the appearance of aging skin, enlarged pores, and acne scars [21]. Park et al. for the first time reported the successful application of FMR to reduce facial erythema in rosacea patients [22]. An observational study demonstrated the ability of FMR to alleviate erythema, flushing, and burning sensation in patients with refractory rosacea [23]. High‐energy FMR, however, is found to cause rosacea‐related symptoms [24].

In the present study, we established a rosacea‐like dermatitis mouse model to identify optimal parameters of FMR to treat rosacea and explored potential mechanisms of action, and validated the efficacy of low‐power short‐pulse‐width fractional microneedle radiofrequency (LS‐FMR) in a cohort of 20 patients with erythematotelangiectatic rosacea (ETR). Our findings are expected to provide valuable references for clinical management of rosacea.

## 2 Methods

### 2.1 A Rosacea‐Like Dermatitis Mouse Model

Female BALB/c mice aged 6–8 weeks and weighing 18–22 g were provided by the Medical School of Nantong University and allowed for a 1‐week habituation. All animal procedures were approved by the Ethics Committee of Nantong University (Ethical No: S20240215‐030). Briefly, LL37, with an amino acid sequence of LLGDFFRKSKEKIGKEFKRIVQRIKDFLRNLVPRTE and a purity of 99% was synthesized by Gill Biotech Co. Ltd. (Shanghai, China), pre‐dissolved in phosphate‐buffered saline (PBS) at a concentration of 320 μM, and stored at 4°C for later use. Twenty‐four hours before the experiment, the dorsal fur of the female BALB/c mouse was shaved. After shaving the dorsal fur, weighing, and anesthetizing, four intradermal injections of 40 μL of LL37 were performed into the back to form a wheal, with an interval of 12 h. Mice in the blank control group were similarly injected with PBS.

In the first part of in vivo experiments, FMR in mice was performed at varying energy levels of 0, 1, 2, 3, 4, 5, 6, and 7 W, but a fixed pulse width of 20 ms at 12 h after the last injection of LL37.

In the second part of in vivo experiments, PBS‐ or LL37‐induced mice were treated with blank control, FMR without releasing energy, and one‐pass or three‐pass FMR (one or three passes of FMR at 2 W and 20 ms at the same site). Non‐insulated needles with a depth of 0.5 mm were used for the thinner skin of mice. At 72 h of FMR, mice were captured and sacrificed to collect skin tissue.

### 2.2 Histopathological Analysis

Mouse skin samples were fixed in 4% formaldehyde solution, embedded in paraffin, and sectioned into 5‐μm slices for staining with hematoxylin and eosin (H&E) or toluidine blue. Images were observed and captured in a blinded way using an optical microscope.

### 2.3 Immunohistochemical and Immunofluorescence Staining

Paraffin‐embedded sections of skin tissue were prepared and incubated overnight at 4°C with anti‐CD4 antibody (1:1000, Abcam, UK), anti‐CD31 antibody (1:2000, Abcam, UK), and the goat anti‐rabbit secondary antibody HRP conjugated (1:50, Beyotime Biotechnology, China) for 1 h at room temperature. After counterstaining with hematoxylin or DAPI, images were observed and captured in a blinded way using an optical microscope or fluorescence microscope.

### 2.4 Quantitative Reverse Transcription Polymerase Chain Reaction (qRT‐PCR)

Total RNA was extracted using TRIzol reagent (Takara, Shiga, Japan) and cDNA was synthesized using the PrimeScript RT Reagent Kit (Takara, Shiga, Japan). TB Green Premix Ex Taq II (Takara, Shiga, Japan) was used for qRT‐PCR on the BIO‐RAD CFX system (BioRad, Munich, Germany). Relative expressions of the target genes were normalized to ACTB using the 2^−ΔΔCt^ method. Primers were designed by Sangon Biotech (Shanghai, China), and their sequences were listed in Data S1.

### 2.5 Western Blotting

Skin tissue was lysed with RIPA lysis buffer (Beyotime, China) to prepare protein samples, whose concentration was quantified using the BCA Protein Assay Kit (Beyotime, China). After SDS‐PAGE, proteins were transferred to PVDF membranes, followed by blocking with 5% BSA in TBST (0.05% Tween 20 in TBS) for 1 h at room temperature. Membranes were then incubated overnight at 4°C with primary antibodies (all from Abcam, UK): anti‐P65 (1:5000), anti‐phospho‐P65 (1:1000), anti‐IL‐1β (1:1000), anti‐IL‐6 (1:1000), anti‐TNF‐α (1:1000), and anti‐β‐actin (1:5000, internal reference). After washing three times with PBS (10 min each) to remove unbound primary antibodies, membranes were incubated with goat anti‐mouse/rabbit HRP‐conjugated secondary antibody (1:1000, Beyotime, China) for 1 h at room temperature. Chemiluminescent imaging was performed on the Tanon 5200 System (Tanon Science&Technology, Shanghai, China). Protein band gray values were quantified via ImageJ (v1.53e), and the relative expression levels of P65, phospho‐P65, IL‐1β, IL‐6, and TNF‐α were normalized to β‐actin.

### 2.6 Study Population

This study was approved by the Ethics Committee of the Third People's Hospital of Nantong (Ethical No: EL2024025). A total of 20 patients diagnosed with ETR independently by two dermatologists according to the Standard Classification and Pathophysiology of Rosacea: The 2017 Update by the National Rosacea Society Expert Committee were recruited [25]. Participants with a history of phototherapy or medication within the previous 3 months, tendency to keloids, and coagulation disorders were excluded.

### 2.7 FMR Procedures

FMR was performed on the same operator (United, Peninsula Medical Treatment Group, Shenzhen, China) consisting of an array of 49 (7 × 7) non‐insulated needles. One hour prior to FMR, compound lidocaine cream was topically applied, followed by routine skin cleansing and disinfection. Parameters of FMR, including the depth (0.9–2.0 mm), pulse energy (6–10 W), and pulse width (60–100 ms), were adjusted based on facial regions, with 20% overlap treatment. Immediately after the procedure, a continuous 1‐hour cold compress was applied to the treated area using sterile medical cooling patches. Furthermore, local yellow light irradiation was administered for 15 min daily over the following three consecutive days to consolidate the anti‐inflammatory effect and promote skin barrier stabilization. All patients received detailed postoperative home care instructions. These included, for the first 7 days post‐treatment, gently rinsing the face only with lukewarm water at 32°C–34°C and avoiding the use of facial cleansers. For the first 14 days, medical device‐grade moisturizing and repair products were to be applied 3 to 4 times daily. Strict physical sun protection, achieved by wearing a wide‐brimmed hat and a medical sunscreen mask, was mandatory for 30 days, and chemical sunscreens were prohibited. Additionally, patients were advised to avoid spicy foods, alcohol, and overheated meals in their diet, as well as to refrain from saunas, hot springs, and strenuous exercise. The use of all non‐medical skincare products and cosmetics was to be suspended for at least 21 days. Clinical images were obtained using a multispectral skin analysis system (Jiangsu Beining Intelligent Technology Development Co. Ltd., China) before and 3 months after FMR. The percentage of erythematous areas was calculated using ImageJ and GraphPad Prism 8.0.

### 2.8 Assessment of the Severity and Quality of Life in ETR Patients

Two experienced dermatologists independently assessed the severity of ETR and the quality of life using the Clinical Erythema Assessment (CEA), the Investigator Global Assessment (IGA), and the Dermatology Life Quality Index (DLQI).

### 2.9 Statistical Analysis

Data were analyzed using GraphPad Prism 8 (GraphPad Software Inc., San Diego, CA, USA). Statistical analyses were performed using *t*‐tests or one‐way analysis of variance (ANOVA). All normally distributed data were presented as mean ± standard deviation (SD). A *p*‐value of less than 0.05 was considered statistically significant.

## 3 Results

### 3.1 Optimal Parameters of FMR for Treating Mice With LL37‐Induced Rosacea‐Like Dermatitis

**FIGURE 1:**
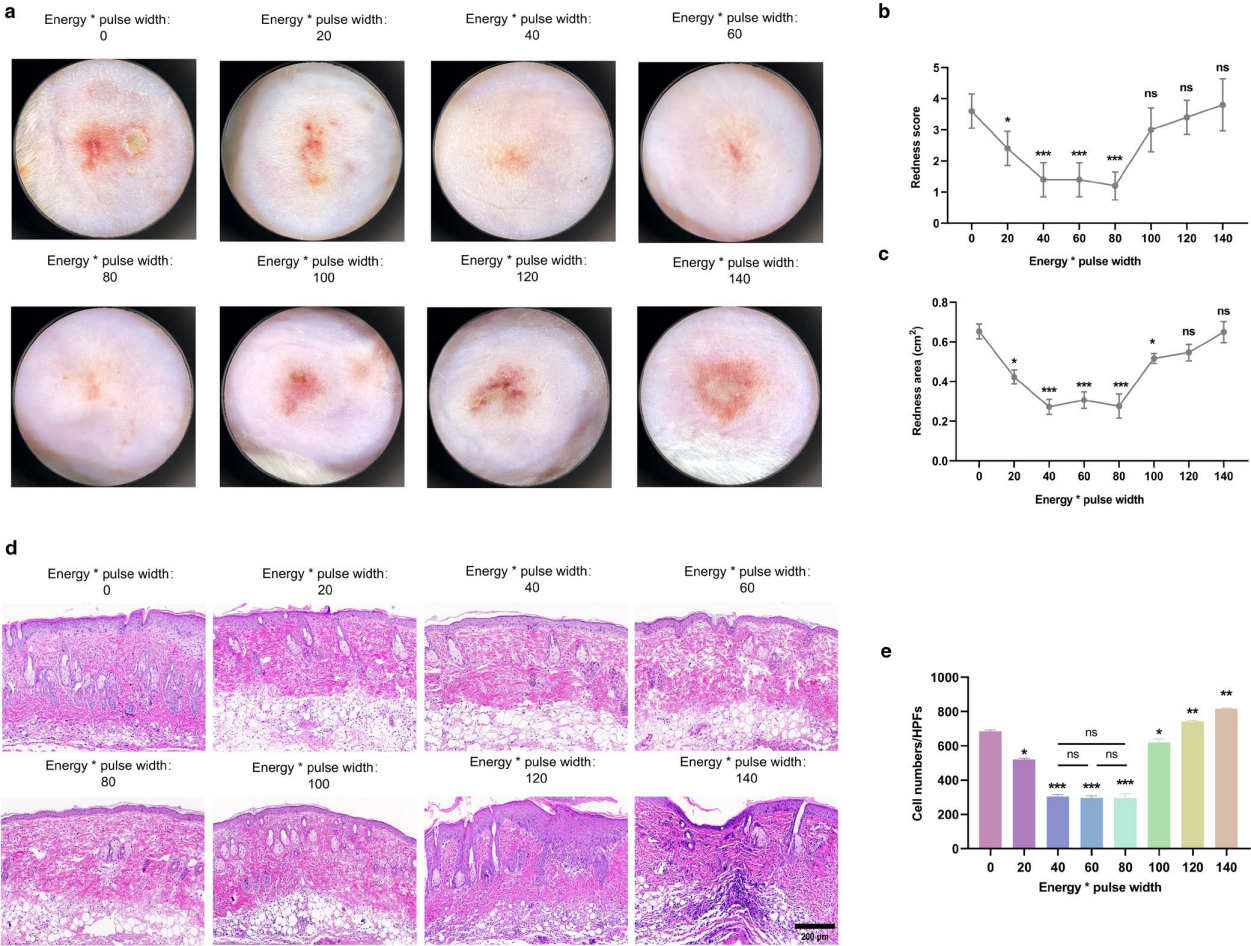
Optimal parameters of FMR to treat LL37‐induced rosacea‐like dermatitis in mice (a) Dermatoscopic images of skin lesions in mice with rosacea‐like dermatitis at 72 h of FMR treatment with varying energy levels (0, 1, 2, 3, 4, 5, 6, and 7 W) and a fixed pulse width (20 ms). (b‐c) Erythema scores (b) and erythema area (c) of mice. (d‐e) H&E staining of inflammatory cells in mouse skin tissue (d) and quantification analysis (e). Scale bar = 200 μm. *p < 0.05, **p < 0.01, ***p < 0.001, *n* = 5.

In mice with LL37‐induced rosacea‐like dermatitis, significant inflammatory erythema and capillary dilation were observed (Figure 1A). After 72 h of FMR at 1 W and 20 ms, both the erythema score and erythema area were slightly reduced (Figure 1B,C). FMR with energy levels of 2, 3, and 4 W and a pulse width of 20 ms significantly alleviated rosacea‐like dermatitis. However, there was no significant difference in efficacy among these three groups. However, FMR at 5 W and 20 ms could only significantly narrow the erythema area. FMR at energy levels of 6 and 7 W and the pulse width of 20 ms did not significantly alleviate rosacea‐like dermatitis in mice.

H&E staining showed a significantly smaller count of inflammatory cells in LL37‐treated mice treated with FMR at 2, 3 and 4 W and 20 ms (Figure 1D,E), but there lacked a significant difference among the three groups. In contrast, the inflammatory cell count significantly increased in LL37‐treated mice treated with FMR at 5–7 W and 20 ms. Although FMR at energy levels of 2, 3, and 4 W with a 20 ms pulse width demonstrated comparable efficacy in alleviating erythema and reducing inflammatory cell infiltration (*p* > 0.05), the energy level of 2 W was selected for all subsequent experiments as the representative LS‐FMR parameter. This decision was based on the principle of using the “lowest effective energy” to achieve the desired therapeutic effect. This approach aligns with the Arndt‐Schultz rule, where low‐level stimuli are beneficial, while higher energies may become inhibitory or damaging.

### 3.2 LS‐FMR Reduces Skin Inflammation in Mice With LL37‐Induced Rosacea‐Like Dermatitis

**FIGURE 2:**
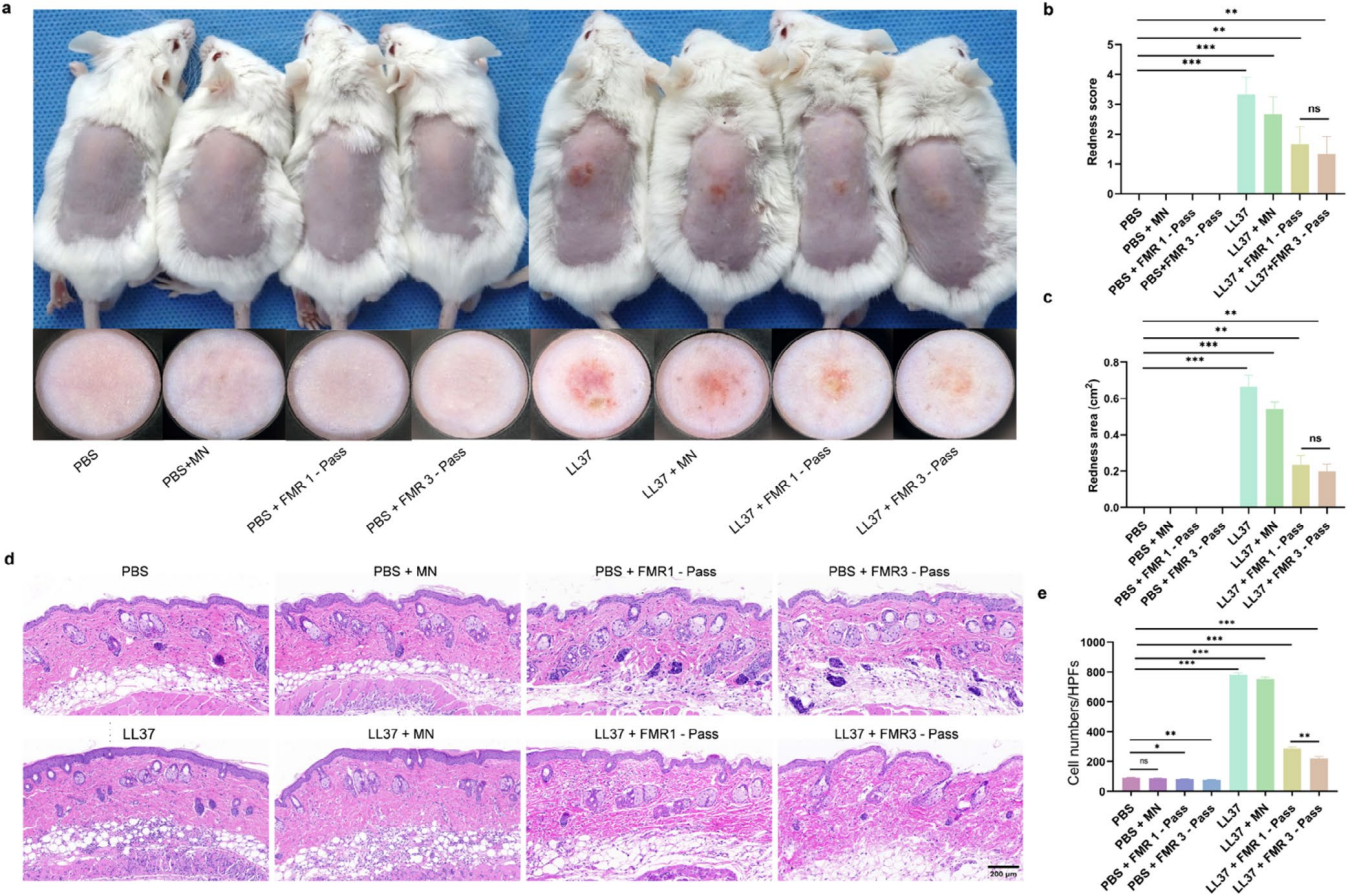
LS‐FMR reduces skin inflammation in mice with LL37‐induced rosacea‐like dermatitis (a) Gross (upper) and dermatoscopic images (bottom) of skin tissue in PBS or LL37‐treated mice treated with FMR without releasing energy, one‐pass or three‐pass LS‐FMR. (b‐c) Erythema scores (b) and erythema area (c) of mice. (d‐e) H&E staining of inflammatory cells in mouse skin tissue (d) and quantification analysis (e). Scale bar = 200 μm. *p < 0.05, **p < 0.01, ***p < 0.001, *n* = 5.

To further validate the efficacy of LS‐FMR on rosacea‐like dermatitis in vivo, PBS or LL37‐treated mice were treated with one‐pass or three‐pass LS‐FMR. Both gross images and dermatoscopic observations that FMR at one‐pass and three‐pass all significantly reduced the erythema scores and erythema area in LL37‐treated mice (Figure 2A–C). The H&E staining results demonstrated that LS‐FMR can significantly reduce inflammatory cell infiltration (Figure 2D,E). Three‐pass FMR presented the most pronounced efficacy in reducing skin inflammation in mice.

**FIGURE 3:**
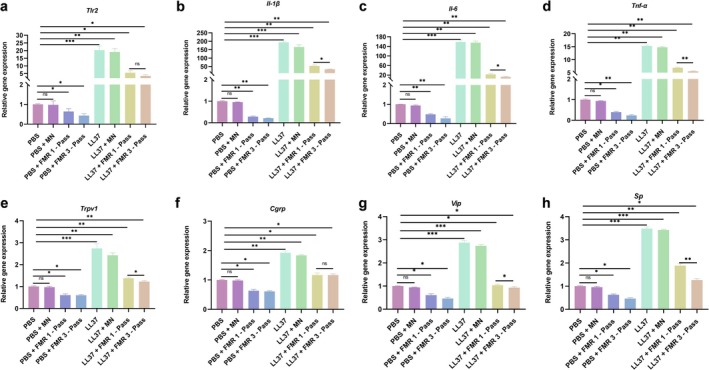
LS‐FMR inhibits pro‐inflammatory factors in mice with LL37‐induced rosacea‐like dermatitis. (a‐h) The mRNA levels of Tlr2 (a), Il‐1β (b), Il‐6 (c), Tnf‐α (d), Trpv1 (e), Cgrp (f), Vip (g), and Sp (h) in skin tissues of PBS or LL37‐treated mice treated with FMR without releasing energy, or one‐pass or three‐pass LS‐FMR. *p < 0.05, **p < 0.01, ***p < 0.001, *n* = 5.

A series of pro‐inflammatory factors in mouse skin tissue were detected by qRT‐PCR (Figure 3A–D). The mRNA levels of TLR2, TNF‐α, IL‐1β, and IL‐6 were all significantly downregulated by LS‐FMR at one‐pass and three‐pass. Except for the mRNA level of TLR2, the efficacy of three‐pass LS‐FMR in lowering pro‐inflammatory factors was superior to one‐pass treatment.

The mRNA levels of TRPV1 and its released neuropeptides, including calcitonin gene‐related peptide (CGRP), vasoactive intestinal peptide (VIP), and substance P (SP), were significantly upregulated in LL37‐treated mice (Figure 3E–H), and then downregulated by one‐pass and three‐pass LS‐FMR significantly. Three‐pass LS‐FMR was more effective than one‐pass LS‐FMR in reducing expression levels of TRPV1, VIP, and SP.

### 3.3 LS‐FMR Inhibits CD4+ T Cell Infiltration and Th1/Th17 Immune Response in Skin Tissue of Mice With LL37‐Induced Rosacea‐Like Dermatitis

**FIGURE 4:**
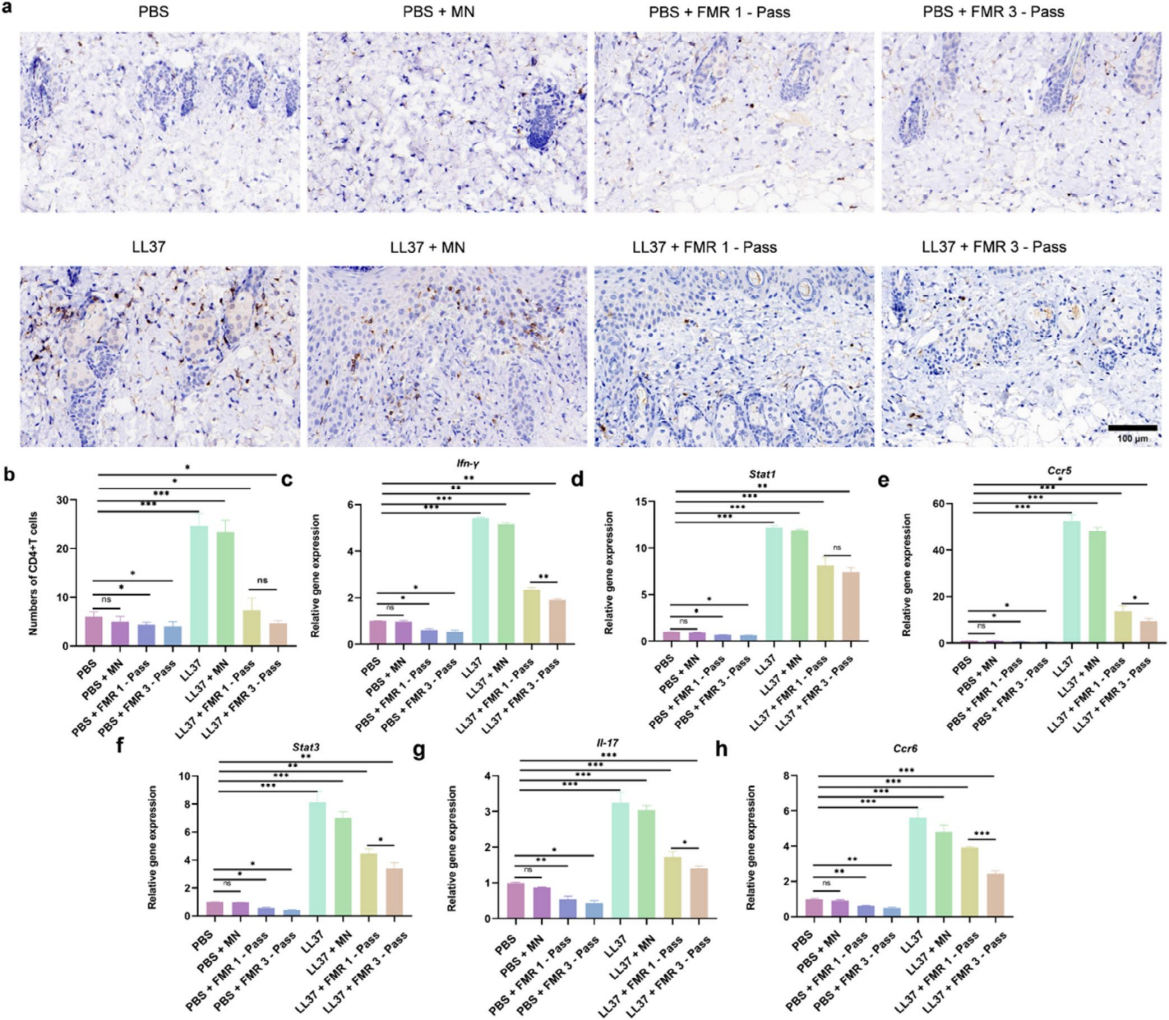
LS‐FMR inhibits CD4^+^ T cell infiltration and Th1/Th17 immune response in skin tissue of mice with LL37‐induced rosacea‐like dermatitis. (a‐b) Immunohistochemical staining of CD4^+^ T cells in skin tissue of PBS or LL37‐treated mice treated with FMR without releasing energy, one‐pass or three‐pass LS‐FMR (a) and quantitation analysis (b). (c‐h) The mRNA levels of Ifn‐γ (c), Stat1 (d), Ccr5 (e), Stat3 (f), Il‐17 (g), and Ccr6 (h) in mouse skin tissue. Scale bar = 100 μm. *p < 0.05, **p < 0.01, ***p < 0.001, *n* = 5.

The number of infiltrating CD4^+^ T cells significantly increased in LL37‐treated mice. Notably, the infiltration of CD4^+^ T cells was significantly suppressed by one‐pass and three‐pass LS‐FMR, showing a comparable outcome between groups (Figure 4A,B).

Expression levels of genes associated with Th1/Th17 polarization were examined using qRT‐PCR. LS‐FMR at one‐pass and three‐pass treatments all significantly downregulated their expression levels (Figure 4C–H). Besides, three‐pass LS‐FMR achieved the most excellent performance in suppressing Th1/Th17 polarization.

### 3.4 LS‐FMR Inhibits Mast Cell Infiltration in Skin Tissue of Mice With LL37‐Induced Rosacea‐Like Dermatitis

**FIGURE 5:**
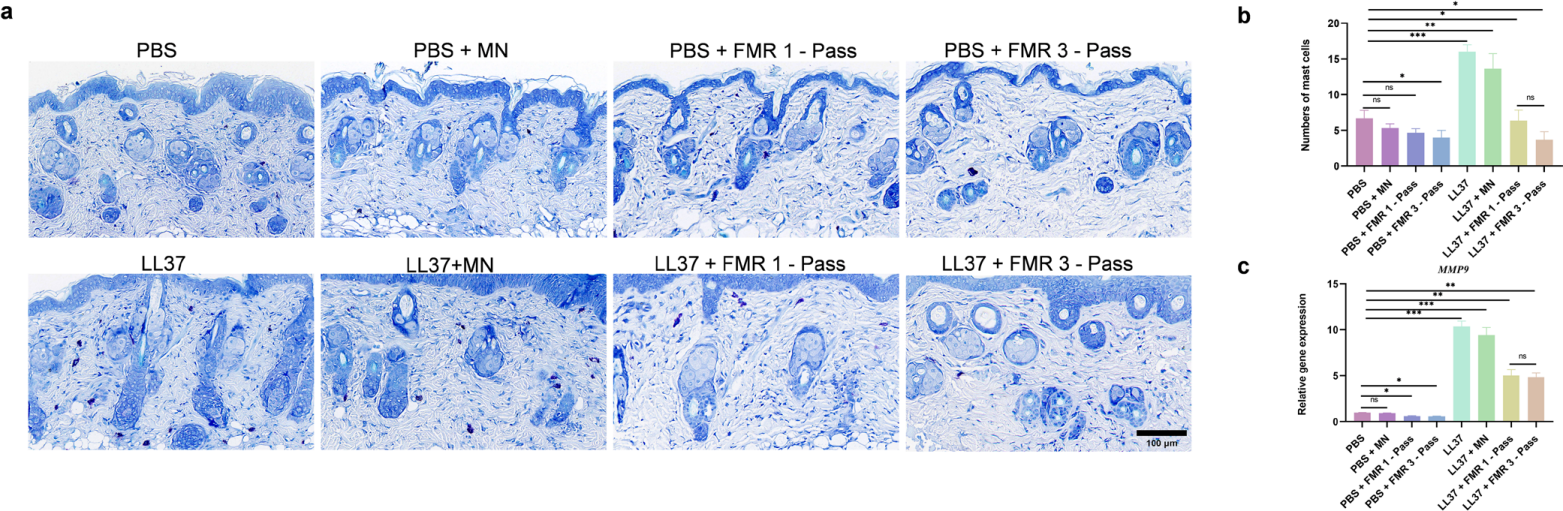
LS‐FMR inhibits mast cell infiltration in the skin tissue of mice with LL37‐induced rosacea‐like dermatitis. (a‐b) Toluidine blue staining of mast cells in skin tissue of PBS or LL37‐treated mice treated with FMR without releasing energy, one‐pass or three‐pass LS‐FMR (a) and quantitation analysis (b). (c) The mRNA level of MMP9 in mouse skin tissue. Scale bar = 100 μm. *p < 0.05, **p < 0.01, ***p < 0.001, *n* = 5.

An obvious mast cell infiltration was found in LL37‐treated mice (Figure 5A,B). Both the LS‐FMR one‐pass and three‐pass treatments significantly inhibited mast cell infiltration in mice with LL37‐induced rosacea‐like dermatitis, presenting a similar outcome between groups. The qRT‐PCR results for MMP9 mRNA align with this finding (Figure 5C).

### 3.5 LS‐FMR Inhibits Angiogenesis in Skin Tissue of Mice With LL37‐Induced Rosacea‐Like Dermatitis

**FIGURE 6:**
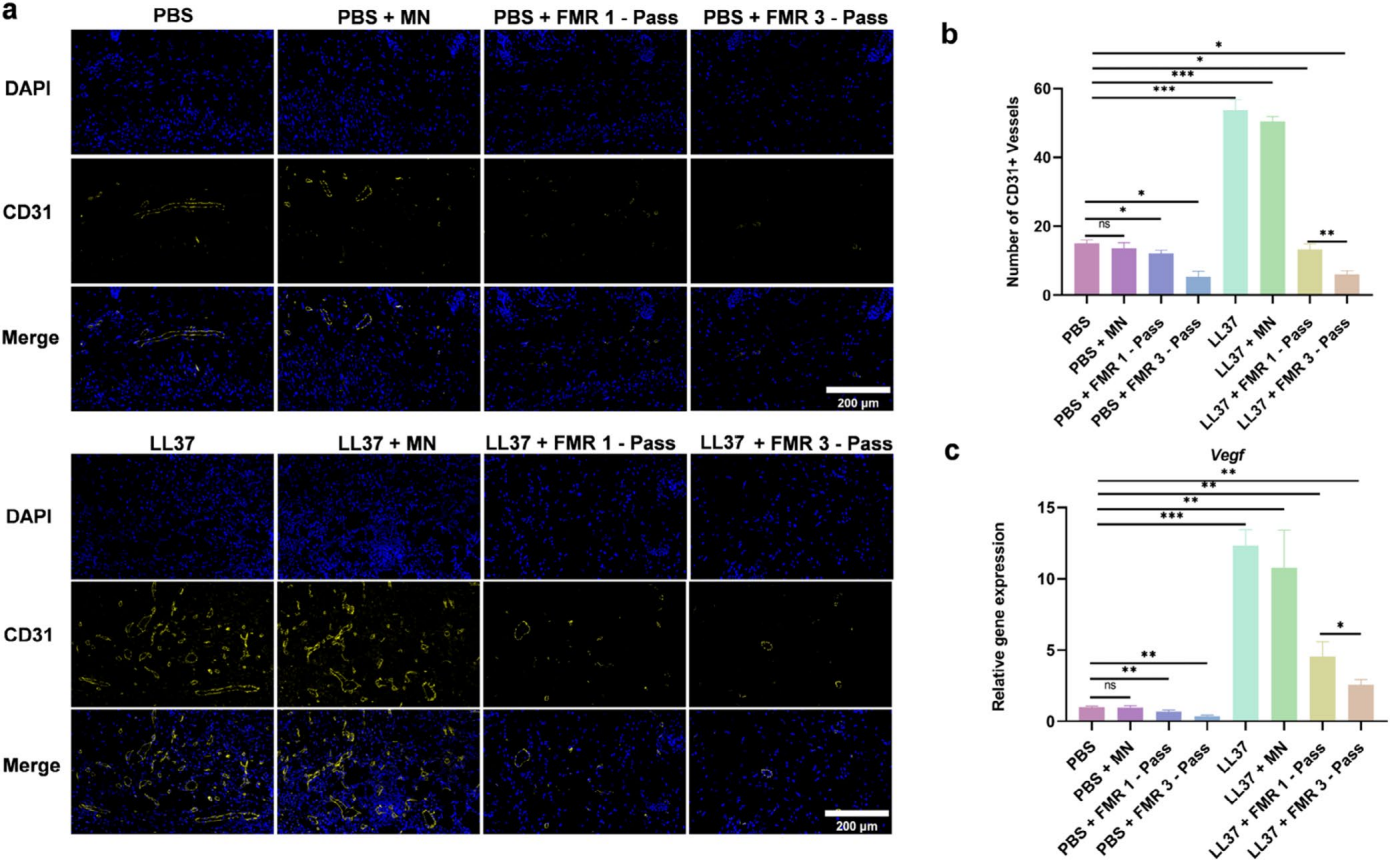
LS‐FMR inhibits angiogenesis in the skin tissue of mice with LL37‐induced rosacea‐like dermatitis. (a‐b) Immunofluorescent staining of CD31 in skin tissue of PBS or LL37‐treated mice treated with FMR without releasing energy, or one‐pass or three‐pass LS‐FMR (a) and quantification analysis (b). (c) The mRNA level of Vegf in mouse skin tissue. Scale bar = 200 μm. *p < 0.05, **p < 0.01, ***p < 0.001, *n* = 5.

Positive staining of CD31 was significant in LL37‐treated mice (Figure 6A,B). Both one‐pass and three‐pass LS‐FMR significantly reduced the number of CD31+ vessels, more prominently in the three‐pass LS‐FMR group. The qRT‐PCR results for mRNA level of vascular endothelial growth factor (VEGF) align with this finding (Figure 6C).

### 3.6 LS‐FMR Alleviates LL37‐Induced Rosacea‐Like Dermatitis in Mice by Inhibiting the NF‐κB Signaling Pathway

**FIGURE 7:**
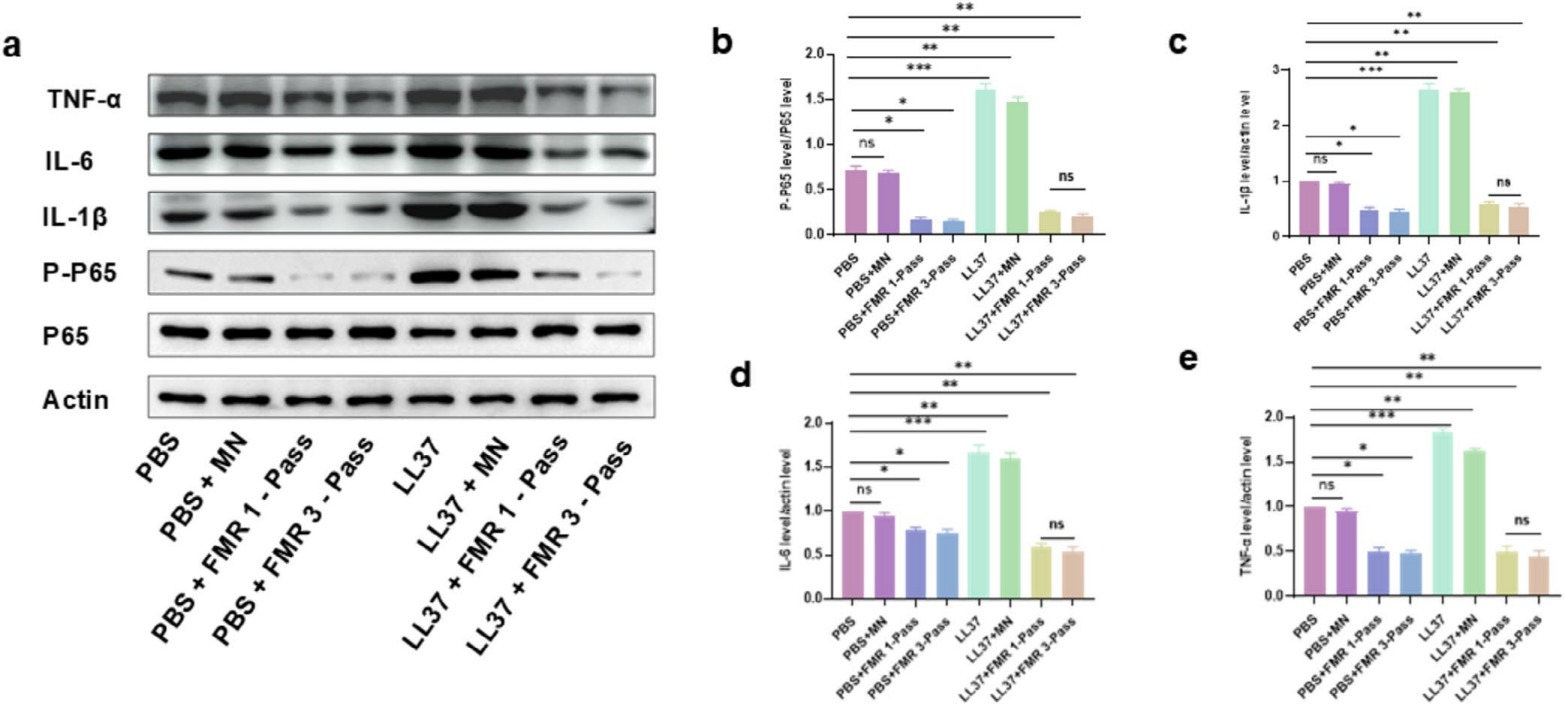
LS‐FMR inhibits the NF‐κB signaling pathway in mice with LL37‐induced rosacea‐like dermatitis. (a‐e) Expression levels of P‐P65, P65, IL‐1β, IL‐6, and TNF‐α in mouse skin tissue of PBS or LL37‐treated mice treated with FMR without releasing energy, one‐pass, or three‐pass LS‐FMR (a) and quantification analysis (b). *p < 0.05, **p < 0.01, ***p < 0.001, *n* = 3.

The protein level of phosphorylated P65 (p‐P65) in the skin tissue of mice with LL37‐induced rosacea‐like dermatitis was significantly upregulated. Both one‐pass and three‐pass LS‐FMR significantly downregulated p‐P65 levels in the skin of LL37‐induced mice (Figure 7A,B). Furthermore, LS‐FMR treatment also markedly suppressed the protein expression of key downstream pro‐inflammatory cytokines, including TNF‐α, IL‐1β, and IL‐6 (Figure 7A–E). This inhibition of the master inflammatory regulator NF‐κB provides direct upstream mechanistic evidence for the concomitant downregulation of its key downstream effector molecules. Collectively, these data indicate that LS‐FMR alleviates skin inflammation in mice, at least in part, by inhibiting the NF‐κB signaling pathway and its mediated cytokine cascade.

### 3.7 Clinical Efficacy of LS‐FMR on Rosacea

**FIGURE 8:**
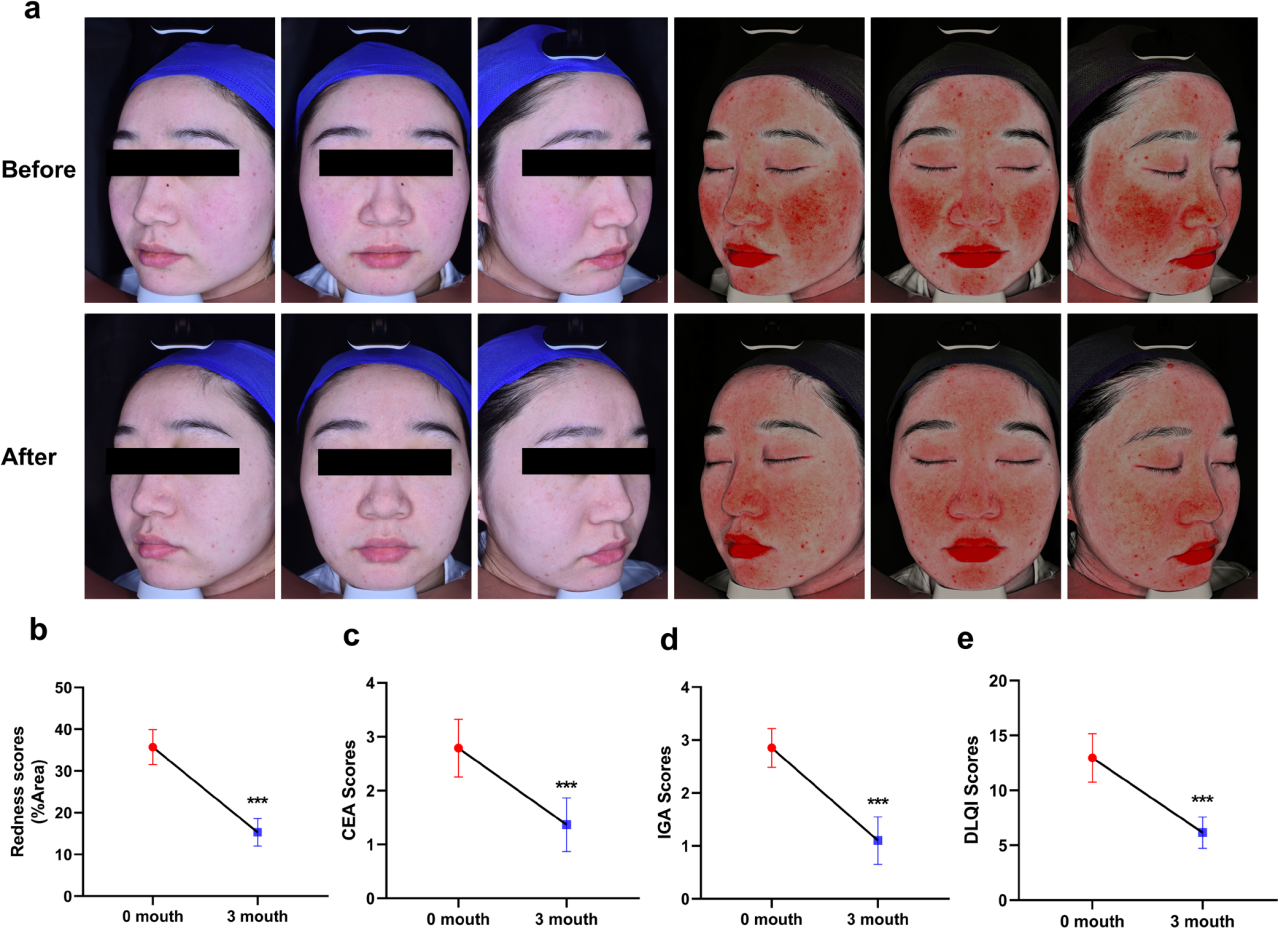
Clinical efficacy of LS‐FMR on rosacea (a) Representative VISIA images of red areas in ETR patients before and 3 months after LS‐FMR, and percentage of red areas (b). Images obtained before treatment and 3 months using a multispectral skin analysis system. (c‐e) Changes in the CEA, IGA, and DLQI scores before and 3 months after LS‐FMR. ***p < 0.001, *n* = 20.

In a cohort of 20 ETR patients treated with LS‐FMR and followed up for 3 months, symptoms of rosacea were significantly alleviated (Figure 8A). In comparison to baseline data, the erythema score (Figure 8B), CEA score (Figure 8C) and IGA score (Figure 8D) at 3 months all significantly decreased, suggesting the severity of ETR reduced by LS‐FMR treatment. Moreover, the DLQI score was significantly reduced at 3 months after LS‐FMR, indicating a better quality of life in ETR patients (Figure 8E), thus validating the clinical efficacy of LS‐FMR on rosacea. Importantly, with the use of optimized LS‐FMR parameters coupled with systematic post‐procedure care, none of the patients experienced exacerbation of erythema or severe adverse events during the follow‐up period. The mild, transient flushing observed immediately after treatment resolved spontaneously within 72 h in all cases, demonstrating the favorable safety and tolerability profile of this treatment approach.

## 4 Discussion

In our study, we first identified the optimal parameters of FMR for treating LL37‐induced rosacea‐like dermatitis and validated the excellent performances of LS‐FMR in reducing skin inflammation by suppressing pro‐inflammatory factors, infiltration of mast cells and CD4^+^ T cells, Th1/Th17 polarization, and the activation of the NF‐κB signaling pathway. Furthermore, our data validated that LS‐FMR provided a treatment protocol in improving the symptoms and quality of life in ETR patients.

Previous evidence has shown the successful application of FMR to skin disorders. Min et al. found that FMR treatment can alleviate acne‐induced inflammatory erythema [26]. Combined with pulsed dye laser and low‐dose isotretinoin, FMR achieves better efficacy on refractory rosacea [27]. Wang et al. reported that lower‐energy short‐wave radiofrequency can significantly relieve symptoms of erythema and capillary dilation in ETR patients. Notably, they avoided applying anesthetics prior to treatment to prevent skin irritation [28]. Our findings consistently demonstrated the efficacy of LS‐FMR in relieving erythema and reducing the erythema area. Non‐insulated needles were used in the present study due to the typical capillary dilation and inflammation in the superficial dermis (the papillary and upper reticular layers) of sensitive skin [29]. Superior to insulated needles that only release radiofrequency heat at the tip, non‐insulated needles can intervene throughout the entire skin layer. However, Aşiran et al. reported a case in which rosacea‐related symptoms appear after dermatological rejuvenation treatment with FMR, which may be attributed to the higher energy and longer pulse width needed to generate greater thermal energy [24]. When the temperature exceeds the threshold of TRPV channels (> 42°C), the TRP channel superfamily gets activated. This triggers neuropeptide release, leading to vascular dysregulation, exacerbated inflammation, and worsening rosacea symptoms [30, 31]. In our study, we also observed irritation on mouse skin followed by FMR treatment at high energy levels (5, 6, and 7 W). A significant reduction in mouse skin inflammation was only observed following an FMR treatment at energy levels of 1, 2, 3, and 4 W and a pulse width of 20 ms. Complying with the Arndt‐Schultz rule, FMR at a small energy level relieved skin inflammation in mice with rosacea‐like dermatitis, while that at moderate‐to‐high energy levels inhibited the therapeutic efficacy of FMR on rosacea [32]. The parameter screening results of this study indicate that selecting lower energy parameters helps prioritize treatment safety, reducing the risk of thermal injury and the possibility of exacerbated inflammation. This choice holds significant strategic importance for clinical translation, as it can maximize the expansion of the therapeutic window and enhance the safety profile of this treatment modality. Seyeon Oh et al. found that low‐energy short‐wave radiofrequency treatment can inhibit the expression levels of TRPV1 and the released neuropeptides in UVB‐induced in vivo and in vitro models, thereby suppressing neuropeptide‐induced inflammatory responses [33]. Similarly, LS‐FMR was found to downregulate TRPV1 and neuropeptides in the rosacea‐like dermatitis mouse model.

Skin immune dysfunction is an established pathogenic mechanism in rosacea [34]. Mast cells are important regulators of skin inflammation in various subtypes of rosacea [35]. Activated mast cells exacerbate the inflammatory response by releasing cytokines and forming a MMP9‐induced positive feedback loop via a self‐amplifying cycle [36]. Treatment with mast cell stabilizers can alleviate rosacea‐related symptoms [37]. The efficacy of intense pulsed light therapy in reducing the inflammatory response in rosacea is related to the inhibition of mast cell degranulation [38]. In our study, LS‐FMR greatly inhibited mast cell activation and downregulated MMP9 in skin tissue of mice with rosacea‐like dermatitis.

Infiltration of CD4^+^ T cells, dominantly by polarized Th1/Th17 cells, is significant in the skin lesions of various subtypes of rosacea [39]. Th1 cells release cytokines like IFN‐γ, enhancing immune activation and inflammation. Th17 cells, a new Th subset, secrete IL‐17, amplifying inflammation through positive feedback [40]. Son M et al., proved the efficacy of radiofrequency treatment on UVB‐induced rosacea‐like lesions in mice through suppressing inflammatory cells and pro‐inflammatory factors [41]. We proposed that in mice with LL37‐induced rosacea‐like dermatitis, LS‐FMR might reduce the production of Th1/Th17‐related cytokines by activating CD4+ T cells. Furthermore, three‐pass LS‐FMR showed superior performances in downregulating Th1/Th17‐related cytokines, neurogenic inflammatory factor TRPV1 and the corresponding neuropeptides than one‐pass treatment. The accumulative outcome of each pass of LS‐FMR eventually resulted in a stronger anti‐inflammatory effect. TLR2 is a receptor that specifically recognizes the components in the innate immune system. Its mRNA level was comparable between the three‐pass and one‐pass LS‐FMR group, suggesting the high safety of multiple sessions of LS‐FMR without additionally activating the skin innate immune system. However, higher treatment frequencies (e.g., 4 or 5 passes) may not necessarily yield better results and could potentially lead to adverse effects. Excessive energy accumulation might overstimulate the skin, disrupt the skin barrier, or even activate TRP channels, exacerbating inflammatory responses and vascular dysregulation.

Besides the excessive activation of inflammatory responses, vascular dilation and abnormal proliferation are also predominant manifestations of rosacea [42]. Traditional phototherapy, such as IPL and PDL, causes a selective thermal effect that generates high energy to damage the abnormal vessels, even though the huge energy may lead to adverse events like purpura and edema [43]. Moreover, IPL and PDL treatments are preferred to small and superficially located vascular dilations [44]. In contrast, energy delivered by FMR penetrates deeper to the dermis without damaging the superlayer structure. Huang et al. demonstrated a significant reduction of facial capillary dilation after a single treatment of FMR in cases of corticosteroid‐induced facial erythema [45]. Our results indicated that LS‐FMR significantly reduced the number of CD31^+^ microvessels in skin lesions of mice with rosacea‐like dermatitis. Therefore, we believe that LS‐FMR directly suppressed newly formed blood vessels.

The NF‐κB signaling pathway has long been recognized for inflammatory mediation in inflammatory skin diseases [46, 47]. Inhibition on the NF‐κB signaling pathway is a promising strategy to treat rosacea [48, 49]. Low‐energy photobiomodulation therapy, a non‐thermal treatment, alleviates inflammation in rosacea mice by inhibiting the NF‐κB signaling pathway [50]. Our data delineate a coherent mechanism of action for LS‐FMR: the suppression of the NF‐κB pathway (evidenced by reduced *p*‐P65) directly leads to the decreased transcription and protein expression of pivotal pro‐inflammatory cytokines such as TNF‐α, IL‐1β, and IL‐6. This axis represents a central pathway through which LS‐FMR alleviates the inflammation of rosacea‐like dermatitis.

Limitations should be noted in the present study. First, mouse skin was thinner than human skin. As a result, optimal parameters of FMR to treat rosacea in mice might not be suitable for human beings. Second, a long‐term follow‐up of LS‐FMR in treating rosacea is required. Third, the subject population was only composed of ETR patients, while patients with other subtypes of rosacea (e.g., papulopustular, ocular, rhinophyma, or neurogenic subtypes) were not taken into consideration. Overall, our findings should be further validated in large‐scale multi‐center clinical trials.

## 5 Conclusion

**FIGURE 9:**
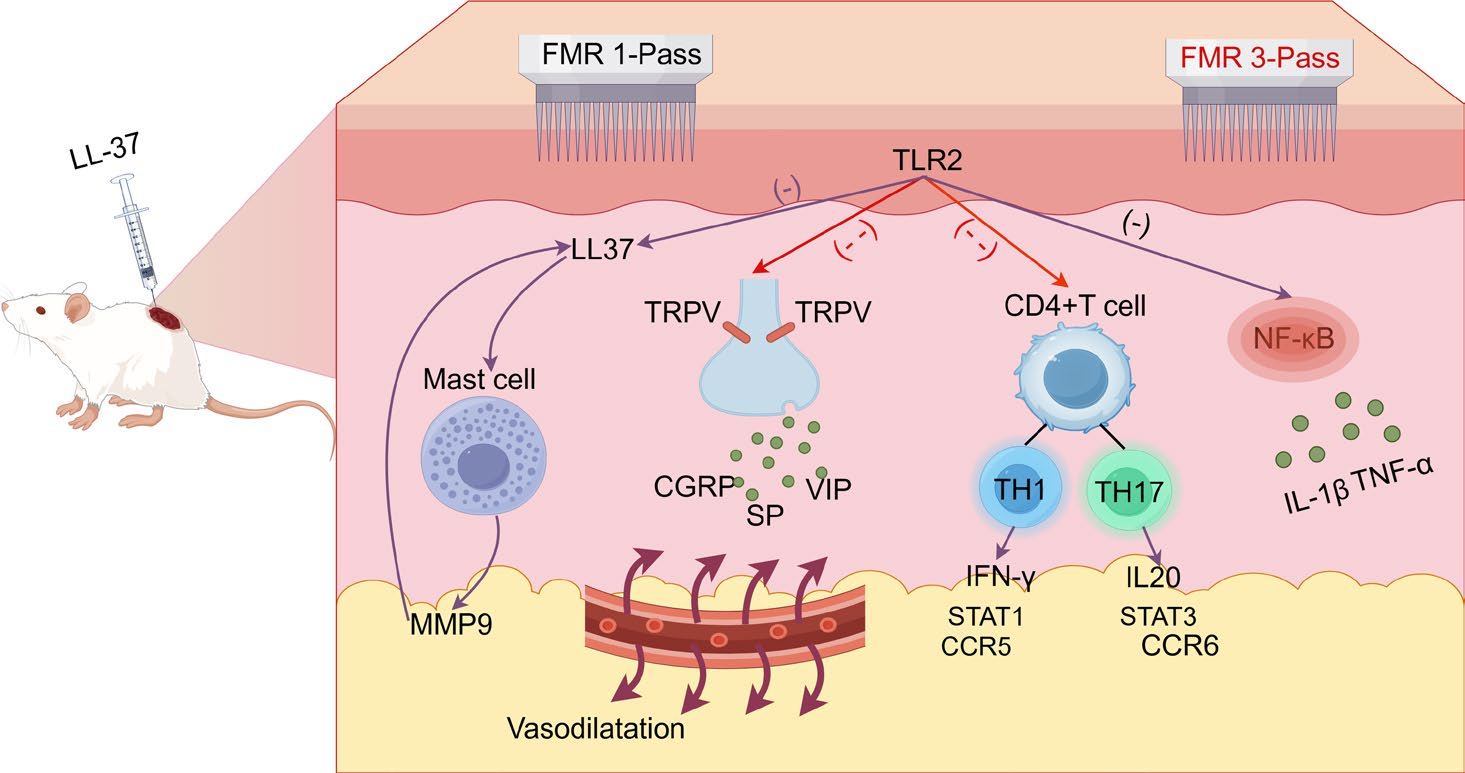
A graphic illustration of LS‐FMR in treating mice with LL37‐induced rosacea‐like dermatitis. Intradermal injections of LL37 on mouse back induced rosacea‐like dermatitis, showing the pathological manifestations of inflammatory and mast cell infiltration, vasodilation due to TRPV activation, polarization of Th1/Th17 cells, and activation of the NF‐κB signaling pathway, which could be significantly inhibited by LS‐FMR. Furthermore, LS‐FMR three‐pass treatment provided a stronger effect than the one‐pass treatment in inhibiting TRPV1, neuropeptides, and Th1/Th17‐related cytokines. By Figdraw.

This study for the first time demonstrated that LS‐FMR alleviates the pathological manifestations of LL37‐induced rosacea‐like dermatitis in mice by inhibiting inflammatory responses, angiogenesis, and the NF‐κB signaling pathway. Furthermore, three‐pass LS‐FMR is superior to one‐pass LS‐FMR in suppressing inflammatory factors, polarization of infiltrating T cells towards Th1/Th17, and angiogenesis (Figure 9). Our findings may provide new strategies for clinical treatment of rosacea‐related dermatitis.

## Author Contributions

Conceived and designed the experiments: Zhiyi Xu, Jingting Zhao, Siqi Shen, Bingrong Zhou, Hui Hua. Performed experiments and analyzed data: Zhiyi Xu, Jingting Zhao, Siqi Shen. Contributed reagents/materials/analysis tool: Xinlan Wang, Jing Wang. Investigation: Li Gu, Shu Zhou, Liqun Gu. Methodology: Lin Chen. Review and editing: Bingrong Zhou, Hui Hua. Writing: Zhiyi Xu, Jingting Zhao. All authors have approved the submission of this manuscript.

## Funding

This work was supported by the National Natural Science Foundation of China (82073472), the Nantong Science Foundation (MS22022102), and the Nantong Young Medical Key Talent Program. Nantong Municipal Health Commission Foundation (MS2023073). Nantong University Special Research Fund for Clinical Medicine (Grant No. 2024JZ021), Nantong University Special Research Fund for Clinical Medicine (Grant No. 2024LQ041) and the Nantong Young Medical Key Talent Program.

## Ethics Statement

The study was conducted in accordance with the Declaration of Helsinki and was approved by the Ethics Committee of Nantong Third People's Hospital (No: EL2024025). All animal procedures were approved by the Ethics Committee of Nantong University (Ethical No: S20240215‐030).

## Consent

Informed consent was obtained from all individual participants included in the study. The authors affirm that human research participants provided informed consent for publication of the images in Figure 8A.

## Conflicts of Interest

The authors acknowledge partial funding for this research from Shenzhen Peninsula Medical Co. Ltd. The sponsor was not involved in the study design; in the collection, analysis, or interpretation of data; in the writing of the manuscript; or in the decision to publish the results. The content is solely the responsibility of the authors.

## Supporting information


**Data S1:** Supporting Information 1.

## Data Availability

The data that support the findings of this study are available on request from the corresponding author. The data are not publicly available due to privacy or ethical restrictions.

## References

[1] T. Searle , F. Al‐Niaimi , and F. R. Ali , “Rosacea,” British Journal of Hospital Medicine (London) 82, no. 2 (2021): 1–8. 10.12968/hmed.2020.0417 33646026

[2] D. Thiboutot , R. Anderson , F. Cook‐Bolden , et al., “Standard Management Options for Rosacea: The 2019 Update by the National Rosacea Society Expert Committee,” Journal of the American Academy of Dermatology 82, no. 6 (2020): 1501–1510. 32035944 10.1016/j.jaad.2020.01.077

[3] H. E. Baldwin , J. Harper , S. Baradaran , and V. Patel , “Erythema of Rosacea Affects Health‐Related Quality of Life: Results of a Survey Conducted in Collaboration With the National Rosacea Society,” Dermatology and Therapy 9, no. 4 (2019): 725–734. 31512178 10.1007/s13555-019-00322-5 PMC6828914

[4] C. Chen , P. Wang , L. Zhang , et al., “Exploring the Pathogenesis and Mechanism‐Targeted Treatments of Rosacea: Previous Understanding and Updates,” Biomedicine 11, no. 8 (2023): 2153. 10.3390/biomedicines11082153 PMC10452301 37626650

[5] M. G. Ivanic , A. Oulee , A. Norden , S. S. Javadi , M. H. Gold , and J. J. Wu , “Neurogenic Rosacea Treatment: A Literature Review,” Journal of Drugs in Dermatology 22, no. 6 (2023): 566–575. 37276164 10.36849/JDD.7181

[6] J. Li , X. Yuan , Y. Tang , et al., “Hydroxychloroquine Is a Novel Therapeutic Approach for Rosacea,” International Immunopharmacology 79 (2020): 106178. 31918061 10.1016/j.intimp.2019.106178

[7] Z. Deng , M. Chen , Y. Liu , et al., “A Positive Feedback Loop Between mTORC1 and Cathelicidin Promotes Skin Inflammation in Rosacea,” EMBO Molecular Medicine 13, no. 5 (2021): e13560. 33734592 10.15252/emmm.202013560 PMC8103105

[8] C. Feng , H. Zhang , P. Wang , et al., “Oroxylin A Suppress LL37 Generated Rosacea‐Like Skin Inflammation Through the Modulation of SIRT3‐SOD2‐NF‐κB Signaling Pathway,” International Immunopharmacology 129 (2024): 111636. 38364746 10.1016/j.intimp.2024.111636

[9] L. Yang , Y. H. Shou , Y. S. Yang , and J. H. Xu , “Elucidating the Immune Infiltration in Acne and Its Comparison With Rosacea by Integrated Bioinformatics Analysis,” PLoS One 16, no. 3 (2021): e0248650. 33760854 10.1371/journal.pone.0248650 PMC7990205

[10] E. Woźniak , A. Owczarczyk‐Saczonek , M. Lange , et al., “The Role of Mast Cells in the Induction and Maintenance of Inflammation in Selected Skin Diseases,” International Journal of Molecular Sciences 24, no. 8 (2023): 7021. 37108184 10.3390/ijms24087021 PMC10139379

[11] F. Cevikbas , X. Wang , T. Akiyama , et al., “A Sensory Neuron‐Expressed IL‐31 Receptor Mediates T Helper Cell‐Dependent Itch: Involvement of TRPV1 and TRPA1,” Journal of Allergy and Clinical Immunology 133, no. 2 (2014): 448–460. 24373353 10.1016/j.jaci.2013.10.048 PMC3960328

[12] S. G. Lee , J. Kim , Y. I. Lee , Y. S. Choi , S. Ham , and J. H. Lee , “Cutaneous Neurogenic Inflammation Mediated by TRPV1‐NGF‐TRKA Pathway Activation in Rosacea Is Exacerbated by the Presence of Demodex Mites,” Journal of the European Academy of Dermatology and Venereology 37, no. 12 (2023): 2589–2600. 37606610 10.1111/jdv.19449

[13] H. Zhang , M. Zhang , Y. Wang , et al., “Murine Models of Rosacea: A Review,” Journal of Cosmetic Dermatology 21, no. 3 (2022): 905–909. 33872453 10.1111/jocd.14164

[14] H. Zhang , K. Tang , Y. Wang , R. Fang , and Q. Sun , “Rosacea Treatment: Review and Update,” Dermatology and Therapy (Heidelberg) 11, no. 1 (2021): 13–24. 10.1007/s13555-020-00461-0 PMC7858727 33170491

[15] U. A. Patil , “Application of Lasers in Vascular Anomalies,” Indian Journal of Plastic Surgery 56, no. 5 (2023): 395–404. 38026771 10.1055/s-0043-1775871 PMC10663075

[16] Y. Wu , Y. Yan , L. Zhang , et al., “Positive Results of Intense Pulsed Light‐Photodynamic Therapy for Moderate‐To‐Severe Rosacea: A Prospective, Single‐Arm Study,” Journal of the American Academy of Dermatology 91, no. 5 (2024): 943–945. 38971190 10.1016/j.jaad.2024.06.063

[17] D. Z. Eichenfield and A. E. Ortiz , “Efficacy and Safety of the 532‐Nm KTP and Long‐Pulsed 1064‐Nm Neodymium‐Doped Yttrium Aluminum Garnet Laser for Treatment of Vascular Malformations,” Dermatologic Surgery 46, no. 12 (2020): 1535–1539. 32371774 10.1097/DSS.0000000000002386

[18] J. Yang , X. Liu , Y. Cao , et al., “5‐Aminolevulinic Acid Photodynamic Therapy Versus Minocycline for Moderate‐To‐Severe Rosacea: A Single‐Center, Randomized, Evaluator‐Blind Controlled Study,” Journal of the American Academy of Dermatology 89, no. 4 (2023): 711–718. 37356626 10.1016/j.jaad.2023.06.027

[19] M. A. Hofmann and P. Lehmann , “Physical Modalities for the Treatment of Rosacea,” Journal der Deutschen Dermatologischen Gesellschaft 14, no. Suppl 6 (2016): 38–43. 10.1111/ddg.13144 27869377

[20] M. Alexiades‐Armenakas , D. Rosenberg , B. Renton , J. Dover , and K. Arndt , “Blinded, Randomized, Quantitative Grading Comparison of Minimally Invasive, Fractional Radiofrequency and Surgical Face‐Lift to Treat Skin Laxity,” Archives of Dermatology 146, no. 4 (2010): 396–405. 20404228 10.1001/archdermatol.2010.24

[21] K. Y. Seo , M. S. Yoon , D. H. Kim , and H. J. Lee , “Skin Rejuvenation by Microneedle Fractional Radiofrequency Treatment in Asian Skin; Clinical and Histological Analysis,” Lasers in Surgery and Medicine 44, no. 8 (2012): 631–636. 22936274 10.1002/lsm.22071

[22] S. Y. Park , H. H. Kwon , J. Y. Yoon , S. Min , and D. H. Suh , “Clinical and Histologic Effects of Fractional Microneedling Radiofrequency Treatment on Rosacea,” Dermatologic Surgery 42, no. 12 (2016): 1362–1369. 27608206 10.1097/DSS.0000000000000888

[23] B. Wang , Y. X. Deng , P. Y. Li , et al., “Efficacy and Safety of Non‐Insulated Fractional Microneedle Radiofrequency for Treating Difficult‐To‐Treat Rosacea: A 48‐Week, Prospective, Observational Study,” Archives of Dermatological Research 314, no. 7 (2022): 643–650. 34196817 10.1007/s00403-021-02259-2

[24] Z. Aşiran Serdar and E. Aktaş Karabay , “A Case of Fractional Microneedling Radiofrequency Induced Rosacea,” Journal of Cosmetic and Laser Therapy 21, no. 6 (2019): 349–351. 31476963 10.1080/14764172.2019.1661487

[25] R. L. Gallo , R. D. Granstein , S. Kang , et al., “Standard Classification and Pathophysiology of Rosacea: The 2017 Update by the National Rosacea Society Expert Committee,” Journal of the American Academy of Dermatology 78, no. 1 (2018): 148–155. 29089180 10.1016/j.jaad.2017.08.037

[26] S. Min , S. Y. Park , J. Y. Yoon , et al., “Fractional Microneedling Radiofrequency Treatment for Acne‐Related Post‐Inflammatory Erythema,” Acta Dermato‐Venereologica 96, no. 1 (2016): 87–91. 26059315 10.2340/00015555-2164

[27] H. H. Kwon , J. Y. Jung , W. Y. Lee , W.‐. Y. Lee , Y. Bae , and G.‐. H. Park , “Combined Treatment of Recalcitrant Papulopustular Rosacea Involving Pulsed Dye Laser and Fractional Microneedling Radiofrequency With Low‐Dose Isotretinoin,” Journal of Cosmetic Dermatology 19, no. 1 (2020): 105–111. 31102325 10.1111/jocd.12982

[28] B. Wang , H. F. Xie , Y. X. Deng , J. Li , and D. Jian , “Efficacy and Safety of Non‐Surgical Short‐Wave Radiofrequency Treatment of Mild‐To‐Moderate Erythematotelangiectatic Rosacea: A Prospective, Open‐Label Pilot Study,” Archives of Dermatological Research 314, no. 4 (2022): 341–347. 33934172 10.1007/s00403-021-02225-y

[29] W. C. Jiang , H. Zhang , Y. Xu , et al., “Cutaneous Vessel Features of Sensitive Skin and Its Underlying Functions,” Skin Research and Technology 26, no. 3 (2020): 431–437. 31793701 10.1111/srt.12819 PMC7317501

[30] S. Bevan , T. Quallo , and D. A. Andersson , “TRPV1,” Handbook of Experimental Pharmacology 222 (2014): 207–245. 24756708 10.1007/978-3-642-54215-2_9

[31] D. Rodrigues‐Braz , M. Zhao , N. Yesilirmak , S. Aractingi , F. Behar‐Cohen , and J. L. Bourges , “Cutaneous and Ocular Rosacea: Common and Specific Physiopathogenic Mechanisms and Study Models,” Molecular Vision 27 (2021): 323–353. 34035646 PMC8131178

[32] D. Mastrangelo , “Hormesis, Epitaxy, the Structure of Liquid Water, and the Science of Homeopathy,” Medical Science Monitor 13, no. 1 (2007): Sr1–Sr8. 17179919

[33] S. Oh , M. Son , J. Park , D. Kang , and K. Byun , “Radiofrequency Irradiation Modulates TRPV1‐Related Burning Sensation in Rosacea,” Molecules 26, no. 5 (2021): 1424. 33800730 10.3390/molecules26051424 PMC7961329

[34] J. W. Marson and H. E. Baldwin , “Rosacea: A Wholistic Review and Update From Pathogenesis to Diagnosis and Therapy,” International Journal of Dermatology 59, no. 6 (2020): e175–e182. 31880327 10.1111/ijd.14757

[35] B. Cribier , “Rosacea Under the Microscope: Characteristic Histological Findings,” Journal of the European Academy of Dermatology and Venereology 27, no. 11 (2013): 1336–1343. 23451732 10.1111/jdv.12121

[36] Y. Muto , Z. Wang , M. Vanderberghe , A. Two , R. L. Gallo , and A. Di Nardo , “Mast Cells Are Key Mediators of Cathelicidin‐Initiated Skin Inflammation in Rosacea,” Journal of Investigative Dermatology 134, no. 11 (2014): 2728–2736. 24844861 10.1038/jid.2014.222 PMC4199909

[37] M. C. Marchitto and A. L. Chien , “Mast Cell Stabilizers in the Treatment of Rosacea: A Review of Existing and Emerging Therapies,” Dermatology and Therapy (Heidelberg) 11, no. 5 (2021): 1541–1549. 10.1007/s13555-021-00597-7 PMC8484408 34476755

[38] P. Jiang , Y. Liu , J. Zhang , et al., “Mast Cell Stabilization: New Mechanism Underlying the Therapeutic Effect of Intense Pulsed Light on Rosacea,” Inflammation Research 72, no. 1 (2023): 75–88. 36329130 10.1007/s00011-022-01635-6

[39] T. T. Brown , E. Y. Choi , D. G. Thomas , A. C. Hristov , and M. P. Chan , “Comparative Analysis of Rosacea and Cutaneous Lupus Erythematosus: Histopathologic Features, T‐Cell Subsets, and Plasmacytoid Dendritic Cells,” Journal of the American Academy of Dermatology 71, no. 1 (2014): 100–107. 24656728 10.1016/j.jaad.2014.01.892

[40] T. Buhl , M. Sulk , P. Nowak , et al., “Molecular and Morphological Characterization of Inflammatory Infiltrate in Rosacea Reveals Activation of Th1/Th17 Pathways,” Journal of Investigative Dermatology 135, no. 9 (2015): 2198–2208. 25848978 10.1038/jid.2015.141

[41] M. Son , J. Park , S. Oh , et al., “Radiofrequency Irradiation Attenuates Angiogenesis and Inflammation in UVB‐Induced Rosacea in Mouse Skin,” Experimental Dermatology 29, no. 7 (2020): 659–666. 32434270 10.1111/exd.14115

[42] J. R. Smith , V. B. Lanier , R. M. Braziel , K. M. Falkenhagen , C. White , and J. T. Rosenbaum , “Expression of Vascular Endothelial Growth Factor and Its Receptors in Rosacea,” British Journal of Ophthalmology 91, no. 2 (2007): 226–229. 17244661 10.1136/bjo.2006.101121 PMC1857639

[43] D. Thaysen‐Petersen , A. M. Erlendsson , J. F. Nash , et al., “Side Effects From Intense Pulsed Light: Importance of Skin Pigmentation, Fluence Level and Ultraviolet Radiation‐A Randomized Controlled Trial,” Lasers in Surgery and Medicine 49, no. 1 (2017): 88–96. 27474536 10.1002/lsm.22566

[44] A. Goldman and U. Wollina , “Complications After Laser Treatment of Facial Vascular Lesions,” Hautarzt 72, no. 5 (2021): 421–425. 33740081 10.1007/s00105-021-04796-3

[45] X. Huang , S. Zheng , P. Chen , et al., “Effective Treatment of Corticosteroid‐Induced Facial Erythema Using Fractional Radiofrequency Microneedling,” Lasers in Surgery and Medicine 56, no. 5 (2024): 466–473. 38693708 10.1002/lsm.23787

[46] M. Corbett , R. Ramessur , D. Marshall , et al., “Biomarkers of Systemic Treatment Response in People With Psoriasis: A Scoping Review,” British Journal of Dermatology 187, no. 4 (2022): 494–506. 35606928 10.1111/bjd.21677 PMC9796396

[47] K. I. Ko , J. J. Merlet , B. P. DerGarabedian , et al., “NF‐κB Perturbation Reveals Unique Immunomodulatory Functions in Prx1(+) Fibroblasts That Promote Development of Atopic Dermatitis,” Science Translational Medicine 14, no. 630 (2022): eabj0324. 35108061 10.1126/scitranslmed.abj0324 PMC8979241

[48] E. J. Wladis , K. W. Lau , and A. P. Adam , “Nuclear Factor Kappa‐B Is Enriched in Eyelid Specimens of Rosacea: Implications for Pathogenesis and Therapy,” American Journal of Ophthalmology 201 (2019): 72–81. 30703356 10.1016/j.ajo.2019.01.018

[49] Q. Zeng , J. Yang , G. Yan , et al., “Celastrol Inhibits LL37‐Induced Rosacea by Inhibiting ca(2+)/CaMKII‐mTOR‐NF‐κB Activation,” Biomedicine & Pharmacotherapy 153 (2022): 113292. 35717785 10.1016/j.biopha.2022.113292

[50] S. Wu , Y. Su , L. Wang , B. Sun , and X. Jiang , “The Effects of Photobiomodulation Therapy on Inflammatory Mediators, Immune Infiltration, and Angiogenesis in a Mouse Model of Rosacea,” Ann Transl Med 10, no. 15 (2022): 831. 36035005 10.21037/atm-22-3204 PMC9403938

